# Prevalence and microbiological and genetic characteristics of multidrug-resistant *Pseudomonas aeruginosa* over three years in Qatar

**DOI:** 10.1017/ash.2022.226

**Published:** 2022-06-20

**Authors:** Mazen A. Sid Ahmed, Hamad Abdel Hadi, Sulieman Abu Jarir, Faisal Ahmad Khan, Mohammed A. Arbab, Jemal M. Hamid, Mohammed A. Alyazidi, Muna A. Al-Maslamani, Sini Skariah, Ali A. Sultan, Abdul Latif Al Khal, Bo Söderquist, Emad Bashir Ibrahim, Jana Jass, Hisham Ziglam

**Affiliations:** 1 Microbiology Division, Department of Laboratory Medicine and Pathology, Hamad Medical Corporation, Doha, Qatar; 2 The Life Science Centre – Biology, School of Science and Technology, Örebro University, Örebro, Sweden; 3 Department of Infectious Diseases, Communicable Diseases Center, Hamad Medical Corporation, Doha, Qatar; 4 College of Art and Science, Qatar University, Doha, Qatar; 5 Department of Microbiology and Immunology, Weill Cornell Medicine – Qatar, Doha, Qatar; 6 School of Medical Sciences, Faculty of Medicine and Health, Orebro University, Orebro, Sweden; 7 Biomedical Research Centre, Qatar University, Doha, Qatar

## Abstract

**Objectives:**

Antimicrobial resistance (AMR) is a global priority with significant clinical and economic consequences. Multidrug-resistant (MDR) *Pseudomonas aeruginosa* is one of the major pathogens associated with significant morbidity and mortality. In healthcare settings, the evaluation of prevalence, microbiological characteristics, as well as mechanisms of resistance is of paramount importance to overcome associated challenges.

**Methods:**

Consecutive clinical specimens of *P. aeruginosa* were collected prospectively from 5 acute-care and specialized hospitals between October 2014 and September 2017, including microbiological, clinical characteristics and outcomes. Identification and antimicrobial susceptibility test were performed using the BD Phoenix identification and susceptibility testing system, matrix-assisted laser desorption ionization–time-of-flight mass spectrometry (MALDI-TOF MS), and minimum inhibitory concentration (MIC) test strips. Overall, 78 selected MDR *P. aeruginosa* isolates were processed for whole-genome sequencing (WGS).

**Results:**

The overall prevalence of MDR *P. aeruginosa* isolates was 5.9% (525 of 8,892) and showed a decreasing trend; 95% of cases were hospital acquired and 44.8% were from respiratory samples. MDR *P. aeruginosa* demonstrated >86% resistance to cefepime, ciprofloxacin, meropenem, and piperacillin-tazobactam but 97.5% susceptibility to colistin. WGS revealed 29 different sequence types: 20.5% ST235, 10.3% ST357, 7.7% ST389, and 7.7% ST1284. ST233 was associated with bloodstream infections and increased 30-day mortality. All ST389 isolates were obtained from patients with cystic fibrosis. Encoded exotoxin genes were detected in 96.2% of isolates.

**Conclusions:**

MDR *P. aeruginosa* isolated from clinical specimens from Qatar has significant resistance to most agents, with a decreasing trend that should be explored further. Genomic analysis revealed the dominance of 5 main clonal clusters associated with mortality and bloodstream infections. Microbiological and genomic monitoring of MDR *P. aeruginosa* has enhanced our understanding of AMR in Qatar.

Over the last several decades, antimicrobial resistance (AMR) has become a global priority because of its substantial clinical and economic impact.^1^ At its forefront, healthcare-associated infections (HAIs) are a major cause of morbidity and mortality in hospitals worldwide. In healthcare settings, the gram-negative ubiquitous opportunistic pathogen *Pseudomonas aeruginosa* is responsible for a wide range of HAIs, including respiratory, urinary, and surgical site infections as well as invasive diseases such as bacteraemia.^2,3^ Emerging resistance of *P. aeruginosa* to different classes of antibiotics is a major concern worldwide. The success of this pathogen is partially due to the multidrug-resistant (MDR) phenotype that *P. aeruginosa* demonstrates,^4^ which more recently has been attributed to the international spread of certain successful clones such as sequence types ST111 and ST235.^5,6^ In addition to multidrug resistance, the ability to form bioﬁlms under different conditions reduces the efﬁciency of the antibiotic treatments and subsequently increases the risk of chronicity.^7^ Moreover, a complex interaction between epidemicity, pathogenicity, regulation, antimicrobial resistance, and the association between mechanisms of resistance and virulence contribute to the severity of *P. aeruginosa*–induced infections.^8^

Globally, the epidemiology of MDR *P. aeruginosa* strains is showing regional variations, but increasing trends ranging from 15% to 30% have been observed across Europe, North America, and South America.^9^ In Europe, isolates of *P. aeruginosa* have become increasingly resistant to standard antipseudomonal therapy, with rates of resistance to fluoroquinolones, piperacillin/tazobactam, carbapenems, ceftazidime, and aminoglycosides exceeding 10%.^10^ Regionally, wide variation in the prevalence of MDR *P. aeruginosa* ranges from low (0%–7.3%), as in Saudi Arabia, to high (50%–75%), as in Egypt, mainly because of heterogeneity of settings and methods of data collection.^11^ The main driver for the global dissemination of resistance is attributed to the spread of high-risk clones, such as ST235, ST111, and ST175, which are associated with HAIs outbreaks and transferable resistance mechanisms, especially horizontally acquired β-lactamases.^12^ Furthermore, *P. aeruginosa* can cause severe infections due to mutations or production of potent virulence factors such as the exotoxins of the type III secretion system (ie, exoS, exoT, exoU, and exoY), which can explain poor clinical outcomes in association with MDR *P. aeruginosa* infections.^13^

Based on local microbiological surveillance and reporting, a high prevalence of MDR Gram-negative bacteria (GNB) isolated from patients at Hamad Medical Corporation (HMC) in Qatar was noted, with *P. aeruginosa* being the second most prevalent (unpublished data). In 2015, 8.1% of *P. aeruginosa* strains from the 5 major hospitals in Qatar were MDR *P. aeruginosa*.^14^ Detailed knowledge regarding characteristics of *P. aeruginosa* circulating in Qatar hospitals is lacking. In this study, we investigated the clinical impact and molecular epidemiology of MDR *P. aeruginosa* in Qatar over a 3-year period.

## Methods

### Study population

In total, 8,892 consecutive *P. aeruginosa* isolates were collected prospectively between October 2014 and September 2017 from various clinical specimens received by the Microbiology Division of the Department of Laboratory Medicine and Pathology (DLMP) from 5 different acute-care and specialized hospitals under HMC, Qatar, as part of the routine care. Specimens were collected from blood, tracheal aspirate, sputum, urine, wound, and corneal swabs. Using a standardized diagnostic and data collection tool, MDR *P. aeruginosa* samples were analyzed and then stored at −80°C pending further analysis. Clinical data were retrospectively collected from the electronic health record. Of 525 MDR *P. aeruginosa* isolates, 78 were selected and subjected to whole-genome sequencing (WGS).

### Study setting and sample collection

During the 3 years from October 2014 to September 2017, a total of 525 MDR *P. aeruginosa* strains were isolated from 273 different patients hospitalized in 5 different HMC hospitals in Doha, Qatar. HMC is the main healthcare provider in the state of Qatar. It serves a population of ∼2.7 million through acute-care and specialized hospitals with a capacity of 1,868 beds and 8 intensive care units in the following facilities: Hamad General Hospital (HGH), an acute-care hospital with 603 beds, Rumaila Hospital for geriatric and long-term patients (RH) with 600 beds, the Women’s Hospital (WH) with 320 beds, the Heart Hospital (HH) with 120 beds, and the National Centre for Cancer and Research (NCCCR) with 46 beds. Patient demographic data, and data related to prior hospitalizations, antibiotic use, clinical diagnosis, risk factors, location of the patients in the hospital, and invasive applications were obtained from the electronic medical record using data-collection forms, with no direct communication between the data collector and patients, primary teams, or the infectious disease team following the patients. *P. aeruginosa* HAIs included signs and symptoms of the infection and species isolation as a unique pathogen for these patients as defined according to the US Center for Disease Control and Prevention (CDC) guidelines.^15^ Colonization was defined as the isolation of *P. aeruginosa* from 2 consecutive cultures of samples from the same site with no evidence of infection. Colonization on admission was defined as the isolation of *P. aeruginosa* within 24 hours. This study was approved by the Research Ethics Committee at Hamad Medical Corporation, Doha, Qatar (protocol no. IRGC-01-51-033).

### The bacterial identification and antimicrobial susceptibility test (ID/AST)

The bacterial identification and initial antimicrobial susceptibility tests (ASTs) of *P. aeruginosa* were performed using the BD Phoenix automated identification and susceptibility testing system (Becton Dickinson, Franklin Lakes, NJ). Identification confirmed was performed using matrix-assisted laser desorption ionization–time-of-flight mass spectrometry (MALDI-TOF MS) on the Bruker Daltonics MALDI Biotyper (Bruker Daltonics, Billerica, MA) according to the manufacturer’s instructions. The minimum inhibitory concentrations (MICs) were determined using Lioﬁlchem MIC test strips (Lioﬁlchem, Rosetodegli Roseto Degli Abruzzi, Italy), while Broth microdilution was used for colistin susceptibility testing (ComASP Colistin, Liofilchem, Roseto degli Abruzzi, Italy). The results were interpreted using Clinical and Laboratory Standards Institute (CLSI) reference break points.^16^ The test was performed for 8 antipseudomonal drugs: gentamicin, tobramycin, amikacin, cefepime, ciprofloxacin, piperacillin-tazobactam, meropenem, and colistin. MDR *P. aeruginosa* isolates were deﬁned as having in vitro nonsusceptibility to at least 1 agent from ≥ 3 antimicrobial classes.^17^

### Genomic assembly, multilocus sequence typing (MLST), phylogenetic tree, and antibiotic resistance genes (ARGs)

In total, 78 MDR *P. aeruginosa* isolates were subjected to WGS using the Illumina HiSeq 2000 system (Illumina, San Diego, CA), performed by Euroﬁns GATC Biotech GmbH, Konstanz, Germany. We applied the following WGS inclusion criteria: MDR *P. aeruginosa* isolated from blood (n = 16), cystic fibrosis patients (n = 12), isolates found to be nonsusceptible to ceftazidime/avibactam and ceftolozane-tazobactam (n = 42), and resistance to all tested antipseudomonal drugs *P. aeruginosa* (n = 8). The clean reads were assembled using SPAdes version 3.13.0 software (Center for Algorithmic Biotechnology, St. Petersburg, Russia).^18^ MLST of MDR *P. aeruginosa* isolates was performed using MLST version 1.8 software (Center for Genomic Epidemiology) based on the 7 housekeeping genes (*acsA*, *aroE*, *guaA*, *mutL*, *nuoD*, *ppsA*, and *trpE*) as described previously.^19^ Annotations generated and detection of exotoxins were performed using the PATRIC RASTtk-enabled Genome Annotation Service.^20^ ARGs were predicted using the Comprehensive Antibiotic Resistance Database (CARD) version 1.2.0 (McMaster University, Hamilton, Ontario).^21^

### Statistical analysis

The data were analyzed in terms of frequency and percentage. The percentages over the years were calculated using the χ^2^ test. To test whether location, acquisition, disease severity, or the number of antibiotic treatments differed over the 3 years, we used the χ^2^ test or the Fisher exact test. For overall mortality, the χ^2^ test or the Fisher exact test was used. A statistically significant difference was considered at *P* < .05 and 95% confidence intervals (CIs) were calculated. Susceptibility patterns of MDR *P. aeruginosa* were determined using frequencies and percentages. Statistical analyses were conducted using IBM SPSS Statistics for Windows version 25.0 software (IBM, Armonk, NY). Heat maps and dendrograms were constructed to visualize and categorize the MDR *P. aeruginosa* based on phenotypic antibiotic resistance, presence of the main exotoxins, mucoidity, important risk factors, and clinical outcomes such as 30-day mortality. The heat maps and dendrograms were constructed in R using gplots function heatmap.2 (R package version 2 software, R Foundation for Statistical Computing, Vienna, Austria). For clustering, the Pearson correlation was used on the presence–absence matrix.^22^

## Results

### Prevalence and distribution of MDR P. aeruginosa isolates

**Fig. 1.**
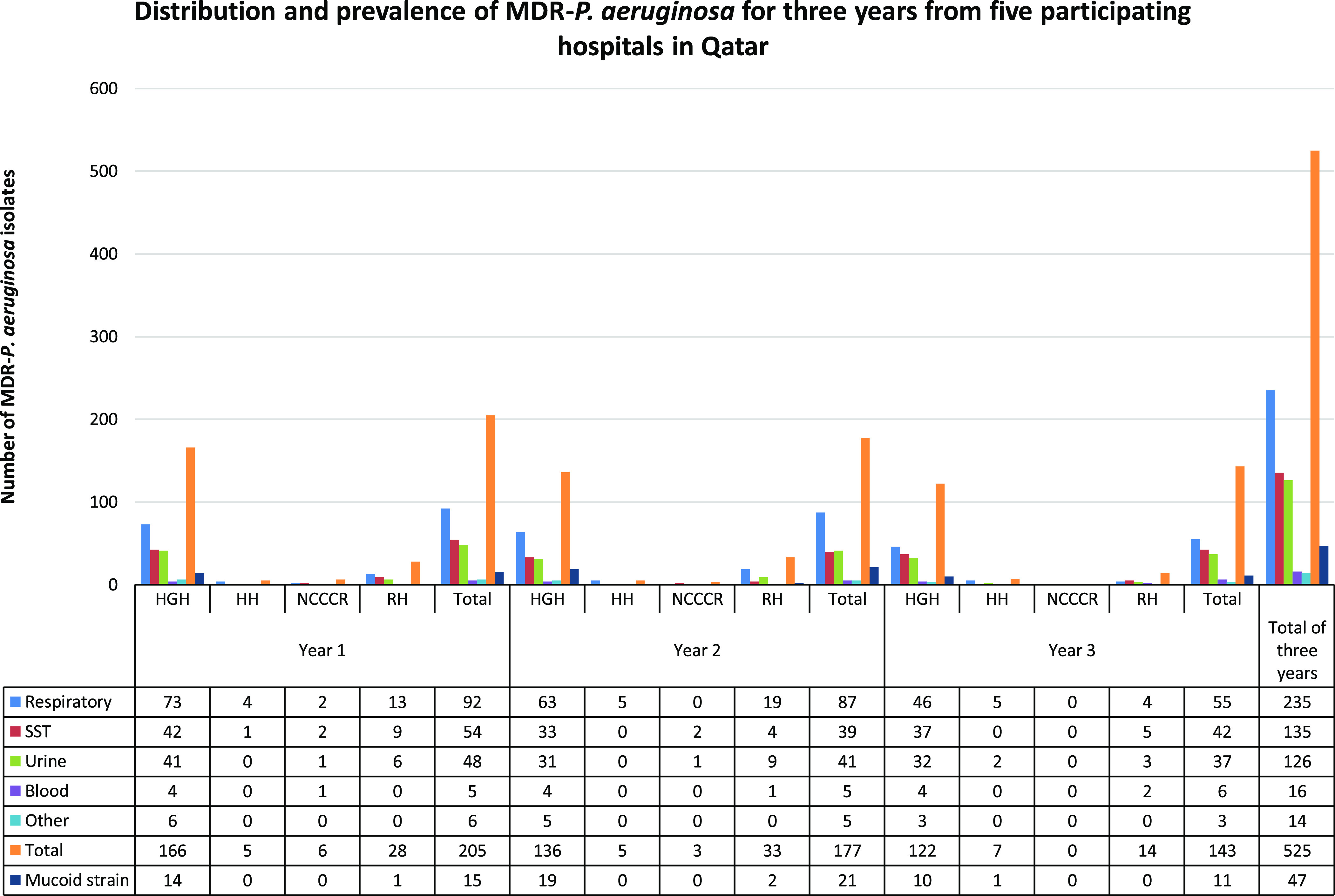
Distribution and prevalence of MDR *P. aeruginosa* for 3 years from 5 participating hospitals in Qatar. Note. HGH, Hamad General Hospital; RH, Rumailah Hospital; NCCCRm National Center for Cancer Care and Research; HH, Heart Hospital; SST, skin and soft-tissue (abscess, biopsy, ear, swab, tissue, and wound); Other, eye, sterile body fluid, nonsterile body fluid, and catheter tip; WH, no MDR *P. aeruginosa* isolates were recovered from the Women’s hospital.

The study included 8,892 consecutive nonduplicate isolates of *P. aeruginosa* (0.6% of 1,054,672 microbiology samples) detected from October 2014 to September 2017 in clinical specimens from patients 5 HMC hospitals (Table 1). The overall prevalence of MDR *P. aeruginosa* was 5.9% (525 of 8,892). The overall prevalence of *P. aeruginosa* isolates did not change significantly over the study period: 0.84% (2,533 of 300,577) in 2015 compared to 0.73% (2,959 of 405,270) in 2017 (Table 1). The 525 MDR *P. aeruginosa* isolates were obtained from 273 different patients. Distributions of MDR *P. aeruginosa* by clinical specimen are shown in percentages for the entire study period (Table 1). Of the 525 isolates, 235 (44.76%) were collected from respiratory samples; 135 (25,71%) were from skin and soft tissue, 126 (24%) were from urine, 16 (3.05%) were from blood, and 30 (5.71%) were from other sample types (Fig. 1).

**Table 1.** Prevalence of Total Samples of *Pseudomonas aeruginosa* Including Multidrug-Resistant Isolates Collected From Five Acute-Care and Specialized Hospitals at Hamad Medical Corporation,^a^ Qatar, Between 2014 and 2017

Period	Total Specimens	Total *P. aeruginosa*	Total number of MDR *P. aeruginosa*	Prevalence of MDR *P. aeruginosa*, %	*P* Value
Oct 2014–Sept 2015	300,577	2,533	205	8.1	<.001
Oct 2015–Sept 2016	348,825	3,400	177	5.2	
Oct 2016–Sept 2017	405,270	2,959	143	4.8	
Total	1,054,672	8,892	525	5.9	

a Hamad Medical Corporation included Hamad General Hospital, Rumailah Hospital, National Center for Cancer Care and Research, Heart Hospital and Women’s Hospital.

*χ^2^ for trend.

### Demographic profile of study population and clinical diagnosis

Overall, 395 MDR *P. aeruginosa* cases (75.2%) were isolated from male patients, aged between 1.5 and 90 years. Overall, 308 patients (58.7%) were aged 14–65 years and 302 patients (57.5%) were non-Qatari nationals. Of 525 cases, 501 (95.4%) were hospital acquired, and 415 (79%) were inpatients (Supplementary Material S1). Furthermore, 298 patients (56.8%) were colonized rather than infected. Sepsis was noted in 122 patients (23.2%) and septic shock occurred in 105 patients (20%). Among 525 MDR *P. aeruginosa* cases, 124 cases (23.6%) were treated with meropenem and 118 (22.5%) were treated with colistin (Supplementary S1). In 88 cases (16.8%), 1 antibiotic was used; in 119 cases (22.7%), a combination of 2 antibiotics was used; in 17 cases (3.2%), 3 antibiotics were used; and in 4 cases (0.8%), 4 antibiotics were used. In 56.8% of colonized cases, however, no antibiotics were used (Supplementary S1).

### Comorbidity factors and clinical outcomes associated with MDR P. aeruginosa infection

**Fig. 2.**
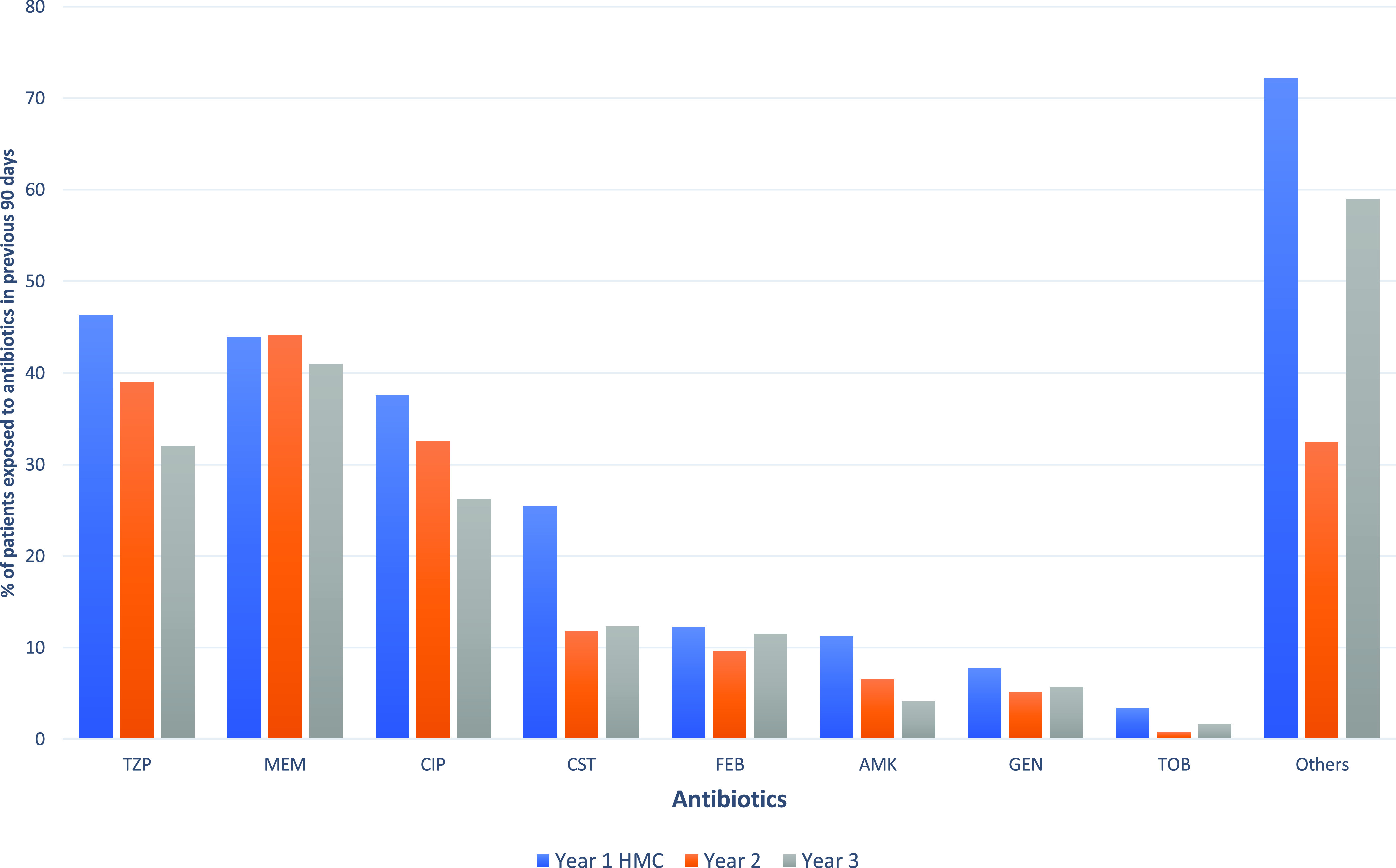
Patient history of previous antibiotic exposure during the prior 90 days from 525 cases with MDR *P. aeruginosa* isolated from Hamad Medical Corporation between October 2014 and September 2017. Antibiotics: AMK, amikacin; CIP, ciprofloxacin; CST, colistin; FEP, cefepime; GEN, gentamicin; MEM, meropenem; TZP, piperacillin/tazobactam; TOB, tobramycin. Others include the following antibiotics: azithromycin, ceftazidime, ceftriaxone, cefuroxime, cefixime, clarithromycin, clindamycin, cloxacillin, doxycyclin, ertapenem, erythromycin, fluroquinolone, levofloxacin, linezolid, meropenem, metronidazole, minocycline, moxifloxacin, nitrofurantoin, rifampcin, septrin, teicoplanin, tigecycline and vancomycin.

The most common risk factors for patients infected with MDR *P. aeruginosa* (Table 2) were extensive healthcare contact (498 cases, 94.9%) and a history of exposure to antibiotics in the previous 90 days (439 cases, 83.6%) (Fig. 2). Other risk factors included presence of invasive devices (349 cases, 66.5%), history of MDR infection or colonization (329 cases, 62.7%), isolation of a prior susceptible strain of *P. aeruginosa* (302 cases, 57.5%), diabetes mellitus (264 cases, 50.3%), and others (Table 2).

**Table 2.** Association of 30 Days Mortality With Patient Characteristics, Site of Infection and Hospital Location With Infections Secondary to MDR *P. aeruginosa* From Hamad Medical Corporation Between October 2014 and September 2017

	Year 1				Year 2				Year 3				Sum of 3 Years				
Characteristics	30-Day Mortality	No Mortality	Total	% of 30-Day Mortality	30-Day Mortality	No Mortality	Total	% of 30-Day Mortality	30-Day Mortality	No Mortality	Total	% of 30-Day Mortality	30-Day Mortality	No Mortality	Total	% of 30-Day Mortality	*P* Value
**Age group**																	
Pediatric, <14 y	0	12	12	0	0	10	10	0	1	13	14	7.1	1	35	36	2.8	.069*
Adult, 14–65 y	5	127	132	3.8	7	98	105	6.7	5	66	71	7.0	17	291	308	5.5	
Geriatric, >65 y	3	58	61	4.9	7	55	62	11.3	10	48	58	17.2	20	161	181	11.0	
**Sex**																	
Male	7	147	154	4.5	12	122	134	9.0	9	98	107	8.4	28	367	395	7.1	.818*
Female	1	50	51	2.0	2	41	43	4.7	7	29	36	19.4	10	120	130	7.7	
**Location**																	
Intensive care unit	7	97	104	6.7	11	55	66	16.7	12	24	36	33.3	30	176	206	14.6	<.001*
Inpatient	1	47	48	2.1	3	29	32	9.4	4	26	30	13.3	8	102	110	7.3	
Outpatient	0	53	53	0	0	79	79	0	0	77	77	0	0	209	209	0	
**Isolation site**																	
Respiratory	4	88	92	4.3	8	79	87	9.2	4	51	55	7.3	16	218	234	6.8	<.001^†^
Skin and soft tissue	2	52	54	3.7	4	35	39	10.3	7	35	42	16.7	13	122	135	9.6	
Urine	0	48	48	0	1	40	41	2.4	1	36	37	2.7	2	124	126	1.6	
Blood	2	3	5	40	1	4	5	20	2	4	6	33.3	5	11	16	31.3	
Sterile body fluid and others	0	6	6	0	0	5	5	0	2	1	3	66.7	2	12	14	14.3	
**Common associated underlying conditions**																	
Extensive healthcare contact^a^	8	182	190	4.2	14	156	170	8.2	16	122	138	11.6	38	460	498	7.6	
History of antibiotic exposure within 90 d	8	167	175	4.6	13	131	144	9	16	104	120	13.3	37	402	439	8.4	
Invasive device^b^	8	134	142	5.6	14	116	130	10.8	15	62	77	19.5	37	312	349	10.6	
Diabetes mellitus	6	91	97	6.2	9	72	81	11.1	13	73	86	15.1	28	236	264	10.6	
History of MDR infection or colonization within prior 90 d	4	120	124	3.2	7	105	112	6.3	12	81	93	12.9	23	306	329	7	
Isolation of prior susceptible *P. aeruginosa*	5	122	127	3.9	9	94	103	8.7	8	64	72	11.1	22	280	302	7.3	
Coinfection with other microorganism^c^	1	51	52	1.9	5	61	66	7.6	8	39	47	17	14	151	165	8.5	
Malignancy	1	34	35	2.9	3	20	23	13	2	14	16	12.5	6	68	74	8.1	
End-stage renal disease	2	26	28	7.1	3	20	23	13	5	26	31	16.1	10	72	82	12.2	
Renal stones	0	20	20	0	0	14	14	0	2	17	19	10.5	2	51	53	3.8	
Heart failure	4	18	22	18.2	2	9	11	18.2	3	16	19	15.8	9	43	52	17.3	
Cystic fibrosis	0	12	12	0	0	6	6	0	0	0	0	0	0	18	18	0	
Chronic obstructive pulmonary disease	2	13	15	13.3	2	19	21	9.5	0	7	7	0	4	39	43	9.3	
Chronic lung disease	1	13	14	7.1	4	44	48	8.3	1	15	16	6.3	6	72	78	7.7	
Post transplantation	0	7	7	0	0	4	4	0	1	1	2	50	1	12	13	7.7	
Chronic liver disease	0	4	4	0	0	0	0	0	1	0	1	100	1	4	5	20	
Neutropenic	0	0	0	0	0	0	0	0	0	1	1	0	0	1	1	0	
Total	8	197	205	3.9	14	163	177	7.9	16	127	143	11.2	38	487	525	7.2	

a Extensive healthcare contact involves regular visits to outpatient medical facilities, regular home visit by home care teams, hospitalization within the preceding 90 days, or residency in a long-term care facility.

b Invasive devices involves exposure to breast implant, central line, colostomy bag, cardiac resynchronization therapy implantable cardioverter defibrillator, double J stent (ureteral stent), external ventricular drain, external fixation of the pelvis, Foley catheter, internal drain, inferior vena cava filter, mechanical ventilator, nephrostomy, nasogastric tube, peritoneal dialysis catheter, permanent pacemaker, right upper abdomen drain, suprapubic catheter, surgical drain, tibial artery stent, tracheotomy tube and ventriculo-peritoneal shunt.

c Coinfection are associated with the following organisms: *Achromoba xylosoxidans*, *Bacteroid fragilis*, *Bacteroid vugatus*, *Escherichia coli*, *Enterococcus fecalis*, *Candida glaberata*, *Candida* spp, *Candida tropicalis*, *Citrobacter froundi*, *Enterobacter cloacae*, *Klebsiella pneumonia*, *K. oxytoca*, *Proteus mirabilis*, methicillin-resistant *Staphylococcus aureus*, methicillin-sensitive *Staphylococcus aureus*, *Serratia marcescenes*, *Stenotrophomonas maltophilia*, *Streptococcus* group C.

* Pearson χ^2^ test of association.

† Fisher exact test.

We analyzed the clinical outcomes of patients infected with MDR *P. aeruginosa.* Overall, 38 (7.2%) of these 525 patients died within 30 days after infection. The mortality rate increased from 3.9% in the first year to 11.2 in the third year, and it was significantly higher in critical care patients compared to other patients (*P* < .001). When clinical outcomes were compared based on isolation of MDR *P. aeruginosa* from different infection sites, we detected a significant association between mortality rate and bacteremia over the 3-year study period of 31.3% (*P* < .001) (Table 2).

### Antimicrobial susceptibility patterns of MDR P. aeruginosa isolates

Over the 3-year study period, the antimicrobial susceptibility results revealed that 97.9% of the clinical isolates were resistant to cefepime, followed by ciprofloxacin (89.5%), meropenem (88.6%), and piperacillin-tazobactam (87%). In addition, the clinical isolates showed less resistance to aminoglycosides such as gentamicin (68%), amikacin (54.9%), and tobramycin (52.8%). However, the isolates were highly susceptible to colistin (2.5% resistance), as shown in the cumulative MIC distribution of antipseudomonal agents (Table 3).

**Table 3.** Cumulative MIC Distribution for Antipseudomonal Agents Against Clinical Isolates of MDR *P. aeruginosa* Between October 2014 and September 2017 From Four Different Hospitals at Hamad Medical Corporation, Qatar

No. (Cumulative %) of Isolates Inhibited at an MIC (mg/L)																						MIC range	Total number of isolates	No. of resistant isolates (%)	Total resistance over three years (%)
Antibiotic	Year/MIC	≤0.75	1	1.5	2	3	4	6	8	12	16	24	32	48	64	96	128	192	256	MIC_50_	MIC_90_				
FEP	Year 1	0 (0)	0 (0)	1 (0.5)	1 (1)	0 (1)	1 (1.5)	0 (1.5)	4 (3.4)	13 (9.8)	16 (17.6)	19 (26.8)	18 (35.6)	17 (44)	13 (50.2)	8 (54.1)	8 (58)	8 (62)	78 (100)	64	256	1.5-256	205	198 (96.6)	513 (97.7)
	Year 2	0	0	0	0	1 (0.6)	0 (0.6)	1 (1.1)	0 (1.1)	8 (5.6)	13 (13)	12 (19.8)	19 (30.5)	13 (37.9)	14 (45.8)	15 (54.2)	6 (57.6)	5 (60.5)	70 (100)	64	256	3-256	177	175 (98.9)	
	Year 3	0	1 (0.7)	0 (0.7)	1 (0.7)	2 (0.7)	3 (0.7)	1 (1.4)	1 (2.1)	6 (6.3)	14 (16.1)	6 (20.3)	12 (28.7)	5 (32.2)	13 (41.3)	5 (44.8)	2 (46.2)	3 (48.3)	74 (100)	256	256	1-256	143	140 (97.9)	
GEN	Year 1	7 (3.4)	7 (6.8)	8 (10.7)	10 (15.6)	13 (22)	9 (26.3)	12 (32.2)	17 (40.5)	1 (41)	6 (44)	6 (46.8)	4 (48.8)	5 (51.2)	2 (52.2)	0 (52.2)	0 (52.2)	3 (53.7)	95 (100)	48	256	0.25-256	205	150 (73.2)	356 (67.8)
	Year 2	8 (4.5)	7 (8,5)	10 (14.1)	10 (19.8)	13 (27.1)	17 (36.7)	9 (41.8)	8 (46.3)	3 (48)	8 (52.5)	2 (53.7)	2 (54.8)	1 (55.4)	2 (56.5)	0 (56.5)	4 (58.8)	0 (58.8)	73 (100)	16	256	0.38-256	177	112 (63.3)	
	Year 3	11 (7.7)	12 (16.1)	9 (22.4)	11 (30.1)	4 (32.9)	2 (34.3)	5 (37.8)	1 (28.5)	4 (41.3)	3 (43.4)	3 (45.5)	2 (46.9)	1 (47.6)	0 (47.6)	2 (49)	0 (49)	0 (49)	73 (100)	256	256	0.5-256	143	94 (65.7)	
TZP	Year 1	0 (0)	2 (1)	0 (1)	1 (1.5)	0 (1.5)	1 (2)	3 (3.4)	3 (4.9)	3 (6.3)	7 (9.8)	10 (14.1)	12 (20)	12 (26.3)	12 (32.2)	9 (36.6)	4 (38.5)	8 (42.9)	118 (100)	256	256	1-256	205	186 (90.7)	457 (87)
	Year 2	1 (0.6)	0 (0.6)	0 (0.6)	3 (2.3)	0 (2.3)	2 (3.4)	1 (4)	1 (4.5)	6 (7.9)	8 (12.4)	8 (16.9)	10 (22.6)	7 (26.6)	7 (30.5)	2 (31.6)	7 (35.6)	1 (36.2)	113 (100)	256	256	0.75-256	177	155 (87.6)	
	Year 3	2 (1.4)	0 (1.4)	0 (1.4)	0 (1.4)	2 (2.8)	2 (4.2)	6 (8.4)	0 (8.4)	4 (11.2)	11 (18.9)	7 (23.8)	13 (32.9)	9 (39.2)	9 (45.5)	8 (51)	8 (56.6)	3 (58.7)	59 (100)	96	256	0.38-256	143	116 (81.1)	
AMK	Year 1	1 (0.5)	1 (1)	2 (2)	2 (2.9)	8 (6.8)	12 (12.7)	8 (16.7)	19 (25.9)	15 (33.2)	16 (41)	13 (47.3)	8 (51.2)	9 (55.6)	6 (58.5)	8 (62.4)	8 (66.3)	2 (67.3)	67 (100)	32	256	0.75-256	205	119 (58.0)	287 (54.7)
	Year 2	0	0	1 (0.6)	5 (3.4)	7 (7.3)	15 (15.8)	10 (21.5)	11 (27.7)	14 (35.6)	10 (41.2)	15 (49.7)	8 (54.2)	9 (59.3)	8 (63.8)	6 (67.2)	7 (71.2)	1 (71.8)	50 (100)	32	256	2-256	177	104 (58.8)	
	Year 3	1 (0.7)	1 (1.4)	3 (3.5)	8 (9.1)	17 (21)	12 (29.4)	12 (37.8)	9 (44.1)	7 (49)	9 (55.2)	9 (61.5)	3 (63.6)	2 (65)	3 (67.1)	1 (67.8)	2 (69.2)	2 (70.6)	42 (100)	16	256	1-256	143	64 (44.8)	
TOB	Year 1	13 (6.3)	20 (16.1)	27 (29.3)	18 (38)	7 (41.4)	7 (44.9)	2 (45.9)	0 (45.9)	4 (47.8)	1 (48.3)	12 (54.1)	13 (60.5)	9 (64.9)	5 (67.3)	4 (69.3)	5 (71.7)	1 (72.2)	57 (100)	24	256	0.38-256	0	112 (54.6)	277 (52.8)
	Year 2	26 (14.7)	19 (25.4)	24 (39)	14 (46.9)	5 (49.7)	6 (53.1)	2 (54.2)	1 (54.8)	0 (54.8)	6 (58.2)	3 (59.9)	8 (64.4)	13 (71.8)	5 (74.6)	1 (75.1)	4 (77.4)	1 (78)	39 (100)	4	256	83 (46.9)	177	83 (46.9)	
	Year 3	43 (30.1)	7 (35)	8 (40.6)	2 (42)	0 (42)	1 (42.7)	4 (45.5)	2 (46.9)	6 (51)	3 (53.1)	7 (58)	2 (59.4)	3 (61.5)	5 (65)	4 (67.8)	4 (70.6)	0 (70.6)	42 (100)	12	256	82 (57.3)	143	82 (57.3)	
CST	Year 1	43 (21)	38 (39)	82 (79.5)	35 (96.6)	3 (98)	2 (99)	1 (99.5)	1 (100)	0 (100)	0 (100)	0 (100)	0 (100)	0 (100)	0 (100)	0 (100)	0 (100)	0 (100)	0 (100)	1.5	2	0.19-8	205	7 (3.4)	13 (2.5)
	Year 2	30 (16.9)	28 (32.8)	76 (75.7)	40 (98.3)	0 (98.3)	1)98.9)	1 (99.4)	1 (100)	0 (100)	0 (100)	0 (100)	0 (100)	0 (100)	0 (100)	0 (100)	0 (100)	0 (100)	0 (100)	1.5	2	0.125-8	177	3 (1.7)	
	Year 3	20 (14)	40 (42)	63 (86)	17 (97.9)	1 (98.6)	0 (98.6)	1 (99.3)	1 (100)	0 (100)	0 (100)	0 (100)	0 (100)	0 (100)	0 (100)	0 (100)	0 (100)	0 (100)	0 (100)	1.5	2	0.125-9	143	3 (2.1)	
MEM	Year 1	8 (3.9)	1 (4.4)	5 (6.8)	6 (9.8)	4 (11.7)	4 (13.7)	4 (15.6)	9 (20)	4 (22)	3 (23.4)	1 (23.9)	156 (100)							32	32	0.125-32	205	185 (90.2)	465 (88.6)
	Year 2	10 (5.60	3 (7.4)	7 (11.3)	1 (11.9)	3 (13.6)	7 (17.5)	7 (21.5)	3 (23.2)	4 (25.4)	2 (26.6)	2 (27.7)	128 (100)	Not applicable						32	32	0.064-32	177	156 (88.1)	
	Year 3	3 (2.1)	4 (4.9)	5 (8.4)	7 (13.3)	2 (14.7)	3 (16.8)	0 (16.8)	0 (16.8)	0 (16.8)	2 (18.2)	1 (18.9)	116 (100)							32	32	0.19-32	143	124 (86.7)	
CIP	Year 1	12 (5.9)	5 (8.3)	5 (10.7)	7 (14.1)	9 (18.5)	14 (25.4)	6 (28.3)	6 (31.2)	6 (34.1)	2 (35.1)	3 (36.6)	130 (100)							32	32	0.125-32	205	187 (91.2)	463 (88.2)
	Year 2	17 (9.6)	5 (12.4)	12 (19.2)	7 (23.2)	9 (28.2)	8 (32.8)	7 (36.7)	5 (39.5)	3 (41.2)	5 (44.1)	0 (44.1)	99 (100)	Not applicable						32	32	0.094-32	177	155 (87.6)	
	Year 3	16 (11.2)	6 (15.4)	5 (18.9)	7 (22.4)	8 (29.4)	8 (35)	6 (39.2)	6 (43.4)	3 (45.5)	3 (47.6)	2 (49)	73 (100)							32	32	0.125-32	143	121 (84.6)	

Note. MIC, minimum inhibitory concentration; AMK, amikacin; CIP, ciprofloxacin; C/T, ceftolozane-tazobactam; CZA, ceftazidime-avibactam; FEP, cefepime; GEN, gentamicin; MDR, multidrug resistant; MEM, meropenem; TZP, piperacillin-tazobactam. No shading indicates susceptible; gray shading indicates nonsusceptible.

### Prevalence distribution, and clustering of the MDR P. aeruginosa

The genomic data analysis showed that the 78 MDR *P. aeruginosa* isolates belonged to 29 different sequence types: 16 ST235 (20.5%), 8 ST357 (10.3%), 6 ST389 (7.7%), 6 ST1284 (7.7%), 6 ST233 (7.7%), and others (36 of 75, 46.2%) (Table 4). Moreover, 9 of 16 ST235 isolates and 2 of 3 ST308 isolates were obtained from patients with urinary tract infections (UTIs) who attended the outpatient department of the acute-care hospital at HGH (*P* < .01). In addition, 5 of 8 ST357 isolates were isolated from the inpatient department at HGH. All 6 ST389 isolates were from cystic fibrosis patients. All 4 ST274 isolates were isolated from patients with respiratory tract infections, and 3 of 5 ST233 isolates were isolated from patients with bloodstream infections. The association of isolation of different sequence types with infection sites and locations is depicted in Table 4.

**Table 4.** The Prevalence and Distribution of Sequence Types of 78 MDR *P. aeruginosa* Isolates Associated With Site of Infection, Location, and Clinical and Microbiological Characteristics

Sequence Type	Isolates, No. (%)	Clinical Diagnosis		Acquisition		Isolation Site					Location			Hospital				Mucoidity	
		Infection	Colonization	Hospital	Community	Respiratory	Skin & Soft Tissue	Urine	Blood	Catheter Tip	OPD	IPD	ICU	HGH	RH	NCCCR	HH	Non-mucoid	Mucoid
ST235	16 (20.5)	10	6	16	0	2	4	9	0	1	9	5	2	16	0	0	1	15	1
ST357	8 (10.3)	6	2	7	1	1	2	3	2	0	2	5	1	8	0	0	0	8	0
ST389	6 (7.7)	3	3	6	0	6	0	0	0	0	5	1	0	6	0	0	0	6	0
ST1284	6 (7.7)	4	2	6	0	0	3	2	1	0	2	3	1	5	1	0	0	5	1
ST233	5 (6.4)	2	3	5	0	1	1	0	3	0	0	4	1	4	1	0	0	4	1
ST274	4 (5.1)	3	1	4	0	4	0	0	0	0	0	1	3	4	0	0	0	3	1
ST244	3 (3.8)	1	2	2	1	0	2	0	1	0	1	0	2	3	0	0	0	3	0
ST308	3 (3.8)	1	2	3	0	1	0	2	0	0	2	1	0	3	0	0	0	1	2
ST823	3 (3.8)	2	1	3	0	2	0	0	1	0	0	3	0	2	0	1	0	3	0
ST2819	2 (2.6)	1	1	2	0	1	0	0	1	0	0	1	1	2	0	0	0	0	2
ST17	1 (1.3)	1	0	1	0	1	0	0	0	0	1	0	0	1	0	0	0	0	1
ST27	1 (1.3)	0	1	1	0	1	0	0	0	0	0	0	1	1	0	0	0	0	1
ST179	1 (1.3)	1	0	1	0	0	1	0	0	0	0	1	0	1	0	0	0	1	0
ST252	1 (1.3)	1	0	1	0	1	0	0	0	0	0	0	1	1	0	0	0	1	0
ST253	1 (1.3)	1	0	1	0	0	1	0	0	0	0	1	0	1	0	0	0	0	1
ST292	1 (1.3)	1	0	1	0	0	0	0	1	0	0	1	0	1	0	0	0	1	0
ST310	1 (1.3)	1	0	1	0	1	0	0	0	0	0	1	0	1	0	0	0	1	0
ST313	1 (1.3)	0	1	1	0	1	0	0	0	0	1	0	0	1	0	0	0	1	0
ST348	1 (1.3)	0	1	1	0	0	0	0	1	0	0	1	0	0	1	0	0	1	0
ST381	1 (1.3)	1	0	1	0	1	0	0	0	0	0	1	0	1	0	0	0	1	0
ST446	1 (1.3)	1	0	1	0	1	0	0	0	0	0	1	0	1	0	0	0	1	0
ST560	1 (1.3)	1	0	1	0	0	1	0	0	0	0	1	0	1	0	0	0	1	0
ST598	1 (1.3)	0	1	1	0	0	0	0	1	0	0	1	0	0	1	0	0	1	0
ST639	1 (1.3)	1	0	1	0	1	0	0	0	0	0	0	1	1	0	0	0	1	0
ST664	1 (1.3)	0	1	1	0	0	1	0	0	0	0	1	0	1	0	0	0	1	0
ST699	1 (1.3)	0	1	1	0	0	0	0	1	0	0	1	0	0	1	0	0	1	0
ST773	1 (1.3)	0	1	1	0	0	0	0	1	0	0	1	0	1	0	0	0	1	0
ST1076	1 (1.3)	1	0	1	0	0	0	0	1	0	0	1	0	1	0	0	0	1	0
ST2178	1 (1.3)	0	1	1	0	1	0	0	0	0	1	0	0	1	0	0	0	1	0
ST3022	1 (1.3)	1	0	1	0	1	0	0	0	0	0	0	1	1	0	0	0	1	0
ST3043	1 (1.3)	0	1	1	0	0	0	0	1	0	0	1	0	1	0	0	0	1	0
ST-Unknown	1 (1.3)	0	1	1	0	1	0	0	0	0	0	1	0	1	0	0	0	1	0
Total (%)	78 (100)	45 (57.7)	33 (42.3)	76 (97.4)	2 (2.6)	29 (37.2)	16 (20.5)	16 (20.5)	16 (20.5)	1 (1.3)	24 (30.8)	39 (50)	15 (19.2)	71 (91)	5 (6.4)	1 (1.3)	1 (1.3)	67 (85.9)	11 (14.1)

Note. HGH, Hamad General Hospital; RH, Rumailah Hospital; NCCCR, National Center for Cancer Care and Research; HH, Heart Hospital; OPD, outpatient department; IPD, inpatient department; ICU, intensive care unit.

**Fig. 3.**
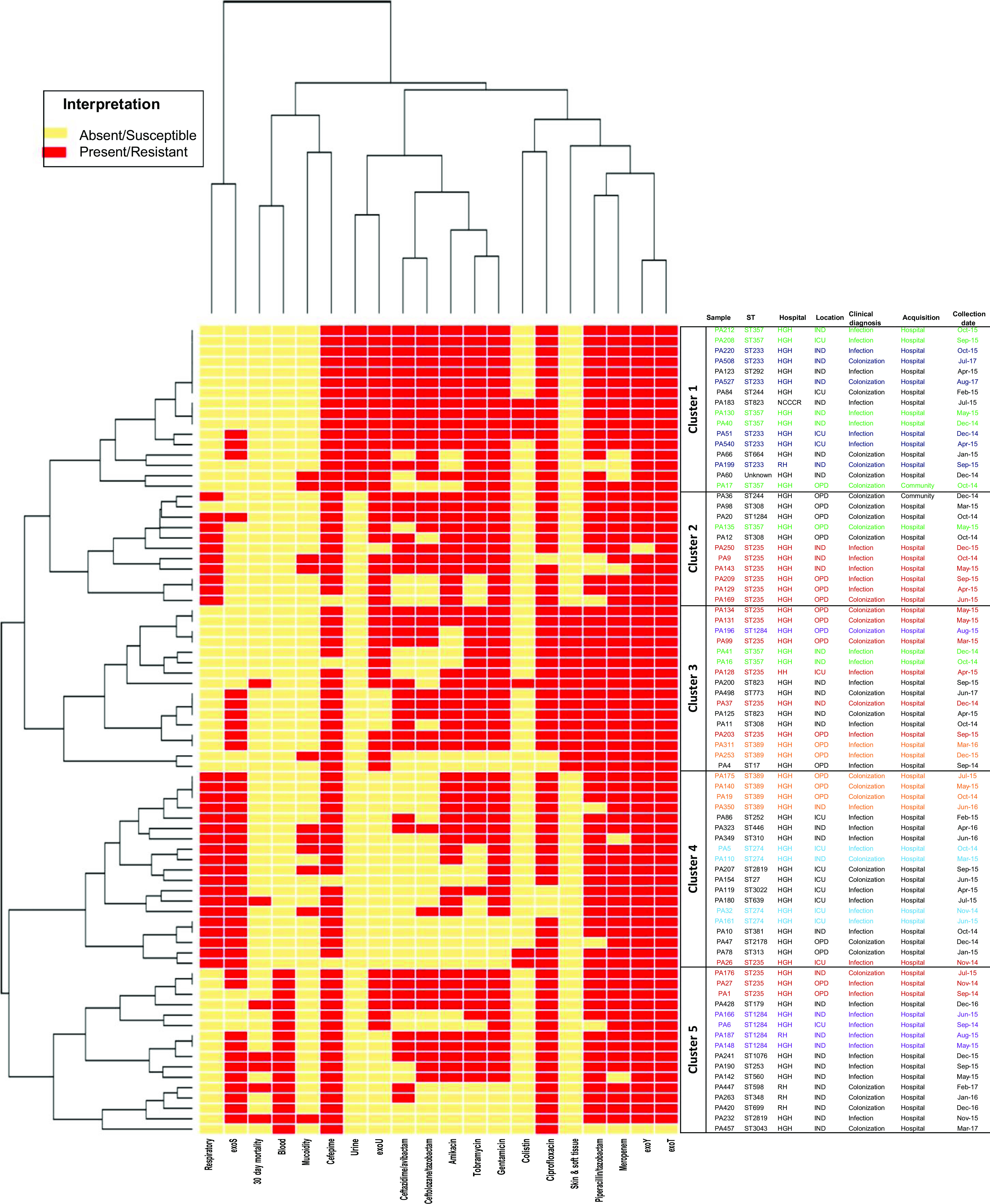
Heat maps and dendograms constructed using the presence or absence of main *P. aeruginosa*–encoded exotoxin, phenotypic resistance to tested antibiotics and important risk factors associated with 78 MDR *P. aeruginosa* isolates collected from Qatar between October 2014 and September 2017. Note. OPD, outpatient department; IPD, inpatient department; ICU, intensive care unit.

Based on phenotypic and genotypic characteristics, important risk factors, and clinical outcomes such as 30-day mortality, we detected 5 clusters (Fig. 3). Cluster analysis of the 78 MDR *P. aeruginosa* showed that cluster 5 was associated with bloodstream infections as well as 30-day mortality (Fig. 3). For the 4 main *P. aeruginosa*–encoded exotoxin genes of the type III secretion system, exoS was detected in 31 (39.7%) of 78 specimens, exoU was detected in 44 specimens (56.4%), exoY was detected in 75 specimens (96.2%), and exoT was detected in 76 specimens (97.4%) (Fig. 3).

## Discussion

The present study is the first and the largest from the Middle East to address systematic surveillance, molecular epidemiology, microbiological and clinical characteristics, as well as clinical outcomes associated with infections secondary to MDR *P. aeruginosa* in a major hospital system. During the 3-year study period in Qatar, the overall prevalence of MDR *P. aeruginosa* was 5.6%, which is lower than reports from neighboring countries or regions.^11^ The prevalence of MDR *P. aeruginosa* initially peaked at 8.1% in the first year of the study, then decreased throughout the study until it reached 4.8% in the third year. At the start the of the study, the upward trend in the epidemiology of GNB, including MDR *P. aeruginosa*, prompted the monitoring process. The significant decline in the prevalence of MDR *P. aeruginosa* over the study period might be attributed to the efficacy of the antimicrobial stewardship program (ASP), which targeted broad-spectrum antimicrobials, including antipseudomonal agents. This effort began around 2015 across all hospitals in Qatar to curb rising AMR rates.^23^ The downward trend of MDR *P. aeruginosa* demonstrates a probable positive impact of the ASP, supported by the observation that the overall proportion of clinical isolates of MDR *P. aeruginosa* did not change over the study period. In addition, we detected a substantial decrease in the use of antipseudomonal agents, reaching 30%, with no major changes in infection control and prevention practices (Table 1).

Similarly, a Cochrane Database systemic review demonstrated the success of ASP interventions to improve antibiotic prescribing in hospital inpatients and reduce AMR.^24^ Our findings agree with those from another study from the United States that investigated the relationship of carbapenem restriction in 22 university teaching hospitals and the incidence of carbapenem-resistant *P. aeruginosa*.^25^

Regarding the isolation of MDR *P. aeruginosa* from different body sites, the distribution and rank order of *P. aeruginosa* isolates were generally in agreement with isolates obtained through the Global SENTRY Antimicrobial Surveillance Program 1997–1999. Respiratory isolates were most prevalent, followed by skin and soft-tissue, urine, and blood isolates.^26^ The low proportion of MDR *P. aeruginosa* bloodstream isolates in this study, which belonged to 13 different sequence types, suggests that cross transmission is not a major problem in the context of MDR *P. aeruginosa* bacteremia in Qatar. Furthermore, 95% of MDR *P. aeruginosa* infections were hospital acquired, and the leading risk factors for acquisition of MDR *P. aeruginosa* included a prior encounter with healthcare settings, colonization with a susceptible *P. aeruginosa* strain, as well as exposure to antimicrobials in the preceding 90 days. These findings are in line with those of previous studies.^14^

Risk factors for acquisition and clinical outcomes for MDR *P. aeruginosa* were not related to age or sex, whereas the 30-day mortality rate significantly increased from 3.9% in the first year to 11.2% in the third year. This rate was significantly higher for critical care patients, and 31.3% of these cases were bloodstream infections (Table 2). This finding is in accordance with previous studies.^27,28^ A noticeable feature of this study was the associated comorbidities with MDR *P. aeruginosa* acquisition (ie, diabetes mellitus, end-stage renal disease, malignancy, heart failure, and chronic lung diseases); however, this observational correlation probably stems from the association of comorbidities with prolonged hospital stay and infection acquisition. Such associations have been observed in other AMR morbidity and mortality studies.^25,29^

Genomic analysis of major clones revealed the dominance of 5 major epidemic sequence types: ST235, ST357, ST389, ST1284, and ST233 (Table 4). These notorious epidemic clones exhibit high MIC values for ceftazidime-avibactam and ceftolozane-tazobactam, and they are responsible for spreading resistance globally, including in the Middle East.^30–32^ Interestingly, most of ST235 and ST308 isolates were isolated from UTIs from patients who attended the outpatient department of the same hospital at HGH, and most ST357 isolates were obtained from patients the inpatient department of the same facility, which supports clonality of HAIs. On the other hand, all ST389 isolates (all from cystic fibrosis patients) and ST274 isolates were obtained from patients with respiratory tract infections, whereas most ST233 isolates were obtained from patients with bloodstream infections. These observations suggest that specific virulence factors are associated with certain sequence types that are site specific.^33,34^ The major clones, dominated by ST235 and ST357, have been associated with global epidemic dissemination and spread of MDR strains. These epidemic clones follow regional locations established at specific healthcare settings, which supports environmental endemicity and clonality of MDR *P. aeruginosa* (Table 4).^22^ Other less frequently isolated clones, such as ST389, ST1284, and ST233, seem to be endemic to our setting. Reports of ST1284 and ST233 are rare and seem to be highly resistant, harboring carbapenem resistance genes such as *bla*_VIM_, which has also been observed in Brazil and Japan.^35,36^ In contrast, none of the previously reported high-risk *P. aeruginosa* clones, such as ST111 and ST175, were detected in the present study.^9^

Our analysis of microbiological characteristics demonstrated a significant resistance profile, reaching or exceeding 90% for primary antipseudomonal agents (Table 3). This observation is worrying because it establishes that AMR for MDR *P. aeruginosa* is a significant challenge with limited treatment options, particularly because other available agents, such as colistin, demonstrated the highest susceptibility rates (97.5%) at the expense of associated adverse events.^37^ Probably because of significant underlying embedded regional resistance profiles, even before the introduction of novel agents such as ceftazidime-avibactam and ceftolozane-tazobactam in our clinical practice, observed susceptibility of the collection for both agents was lower than other regions at 68.8% and 62.9%, respectively.^38^ In our previous study, we examined β-lactamase–mediated genes through selective genomic analysis of 75 MDR *P. aeruginosa* isolates, which included 42 isolates that were resistant to 1 or both agents. They all possessed class C and/or class D β-lactamases; 50% of isolates possessed class A β-lactamases; and 26.7% possessed class B β-lactamases.^39,40^

Additionally, genomic analyses of virulence factors in previous studies have detected the presence of mutations in distinct genes for MDR *P. aeruginosa*–encoded type III exotoxins: exoS, exoT, exoU, exoY, which are known to be associated with severe infections.^13^ In this study, comparative analysis of the presence or absence of *P. aeruginosa* virulence factors against sequence types, site of isolation, antipseudomonal agents, and clinical outcomes revealed that they are clustered within certain sequence types and are related to negative clinical outcomes, such as bloodstream infections, but are probably not related to AMR (Fig. 3).

In conclusion, our 3-year study of 525 clinical isolates of MDR *P. aeruginosa* is one of the largest global collections that established the initial relatively higher prevalence of resistant clinical isolates demonstrating significant AMR in Qatar, which was later ameliorated through effective surveillance, monitoring, and implementation of a successful ASP. We also observed the role of specific endemic clones that were geographically located demonstrating high-level resistance as well as being associated with specific infections such as bloodstream and exacerbation of infections in patients with cystic fibrosis probably related to certain underlying virulence factors. In the fight to control HAIs, we advocate continuous surveillance, monitoring as well as genomic analysis to understand pathogens epidemiology, microbiological characteristics as well as underlying mechanisms of virulence and resistance.

## Supplementary material

For supplementary material accompanying this paper visit https://doi.org/10.1017/ash.2022.226. click here to view supplementary material
