# Supplementary material for: Prevalence and microbiological and genetic characteristics of multidrug-resistant Pseudomonas aeruginosa over three years in Qatar
Source: Antimicrob Steward Healthc Epidemiol. 2022 Jun 20;2(1):e96. doi: 10.1017/ash.2022.226 (PMC9726487; doi:10.1017/ash.2022.226)
Supplement: Supplementary file 1 [file S2732494X22002261sup001.docx]

***Supplementary S1:*** *Three years comparison of clinical diagnosis, treatment and location of patients with MDR-P. aeruginosa infection isolated between October 2014 – September 2017 from 5 different hospitals under Hamad Medical Corporation****.***

| **Characteristics** | **Year 1** | **Year 2** | **Year 3** |  |  |
| --- | --- | --- | --- | --- | --- |
|  | **Frequency (%)** | | | **Total (%)** | ***P*-value** |
| **Location** |  |  |  |  |  |
| Outpatient | 48 (23.4) | 32 (18.1) | 30 (21) | 110 (21) | <0.001 * |
| Inpatient | 104 (50.7) | 66 (37.3) | 36 (25.2) | 206 (39.2) |  |
| Inpatient ICU | 53 (25.9) | 79 (44.6) | 77 (53.8) | 209 (39.8) |  |
| Stay in ICU >5 days | 50 (24.4) | 78 (44.1) | 72 (50.3) | 200 (38.1) |  |
| **Acquisition** |  |  |  |  |  |
| Hospital | 195 (95.1) | 172 (97.2) | 134 (93.7) | 501 (95.4) | 0.324 * |
| Community | 10 (4.9) | 5 (2.8) | 9 (6.3) | 24 (4.6) |  |
| **Disease severity** |  |  |  |  |  |
| Colonization | 121 (59) | 97 (54.8) | 80 (55.9) | 298 (56.8) | 0.9 * |
| Sepsis | 44 (21.5) | 45 (25.4) | 33 (23.1) | 122 (23.2) |  |
| Septic shock | 40 (19.5) | 35 (19.8) | 30 (21) | 105 (20) |  |
| **Antibiotic treatment** |  |  |  |  |  |
| Meropenem | 62 (30.2) | 34 (19.2) | 28 (19.6) | 124 (23.6) |  |
| Colistin | 53 (25.9) | 34 (19.2) | 31 (21.7) | 118 (22.5) |  |
| Piperacillin/tazobactam | 12 (5.9) | 14 (7.9) | 13 (9.1) | 39 (7.4) |  |
| Amikacin | 13 (6.3) | 14 (7.9) | 8 (5.6) | 35 (6.7) |  |
| Ciprofloxacin | 6 (2.9) | 9 (5.1) | 10 (7) | 25 (4.8) |  |
| Cefepime | 4 (2) | 13 (7.3) | 5 (3.5) | 22 (4.2) |  |
| Gentamicin | 6 (2.9) | 9 (5.1) | 3 (2.1) | 18 (3.4) |  |
| Aztreonam | 0 | 2 (1.1) | 6 (4.2) | 8 (1.5) |  |
| Tobramycin | 0 | 1 (0.6) | 0 | 1 (0.2) |  |
| Ceftazidime | 0 | 1 (0.6) | 0 | 1 (0.2) |  |
| **Number of antibiotic treatment(s)** | | | | |  |
| 0 | 121 (59) | 97 (54.8) | 80 (55.9) | 298 (56.8) | 0.19 † |
| 1 | 23 (11.2) | 38 (21.5) | 27 (18.9) | 88 (16.8) |  |
| 2 | 51 (24.9) | 36 (20.3) | 32 (22.4) | 119 (22.7) |  |
| 3 | 7 (3.4) | 7 (4) | 3 (2.1) | 17 (3.2) |  |
| 4 | 3 (1.5) | 0 | 1 (0.7) | 4 (0.8) |  |
| **Total** | 205 (100) | 177 (100) | 143 (100) | 525 (100) |  |

*ICU; Intensive care unit*

** Chi-square test for independence (chi-square test for association)*

*† Fisher's Exact test*
